# Effects of Temperature Adaptation on the Metabolism and Physiological Properties of Sturgeon Fish Larvae Cell Line

**DOI:** 10.3390/cells13030269

**Published:** 2024-01-31

**Authors:** Philipp Lutze, Julia Brenmoehl, Stephanie Tesenvitz, Daniela Ohde, Heike Wanka, Zianka Meyer, Bianka Grunow

**Affiliations:** 1 Fish Growth Physiology, Research Institute for Farm Animal Biology (FBN), 18196 Dummerstorf, Germany; lutze@fbn-dummerstorf.de; 2 Institute of Pathophysiology, University Medicine Greifswald, 17489 Greifswald, Germany; stephanie.tesenvitz@med.uni-greifswald.de; 3 Signal Transduction, Research Institute for Farm Animal Biology (FBN), 18196 Dummerstorf, Germany; brenmoehl@fbn-dummerstorf.de (J.B.); ohde@fbn-dummerstorf.de (D.O.); zianka.meyer@diagenom.de (Z.M.); 4 Institute of Physiology, University Medicine Greifswald, 17489 Greifswald, Germany; heike.wanka@med.uni-greifswald.de; 5 Diagenom GmbH, 18059 Rostock, Germany

**Keywords:** *Acipenser oxyrinchus*, climate change, temperature increase, heat shock, Seahorse XF96, fish cell line

## Abstract

This study investigated how Atlantic sturgeon cells respond to elevated temperatures, shedding light on the potential impacts of climate change on fish. Atlantic sturgeon (*Acipenser oxyrinchus*), an IUCN (International Union for Conservation of Nature) Red List species and evolutionarily related to paleonisiform species, may have considerable physiological adaptability, suggesting that this species may be able to cope with changing climatic conditions and higher temperatures. To test this hypothesis, the AOXlar7y cell line was examined at 20 °C (control) and at elevated temperatures of 25 °C and 28 °C. Parameters including proliferation, vitality, morphology, and gene expressions related to proliferation, stemness, and stress were evaluated. Additionally, to achieve a comprehensive understanding of cellular changes, mitochondrial and metabolic activities were assessed using Seahorse XF96. AOXlar7y cells adapted to 28 °C exhibited enhanced mitochondrial adaptability, plasticity, heightened cell proliferation, and increased *hsp70* expression. Increased baseline respiration indicated elevated ATP demand, which is potentially linked to higher cell proliferation and heat stress defense. Cells at 28 °C also displayed elevated reserve respiration capacity, suggesting adaptation to energy demands. At 25 °C, AOXlar7y cells showed no changes in basal respiration or mitochondrial capacity, suggesting unchanged ATP demand compared to cells cultivated at 20 °C. Proliferation and glycolytic response to energy requirements were diminished, implying a connection between glycolysis inhibition and proliferation suppression. These research results indicate sturgeon cells are capable of withstanding and adapting to an 8 °C temperature increase. This cellular analysis lays a foundation for future studies aimed at a deeper understanding of fish cell physiological adaptations, which will contribute to a better knowledge of environmental threats facing Atlantic sturgeon and fish populations amid climate change.

## 1. Introduction

The Atlantic sturgeon (*Acipenser oxyrinchus*, Mitchill, 1815), a long-standing species within the evolutionary timeline, is currently classified as “possibly extinct” in Europe on the IUCN Red List. This classification stems from the detrimental effects of extensive overfishing, dam construction, river regulation, and pollution, all of which have a major impact on the population. Given these circumstances, there is a concerted effort to reintroduce the Atlantic sturgeon in order to restore its vital role within the ecosystems of northern and central Europe [1,2]. Notably, the species has been bred in a controlled manner in aquaculture facilities for over two decades to strengthen the sturgeon population in the Baltic Sea. Juvenile individuals of this anadromous fish species are primarily released in the Odra and Vistula rivers, their natural spawning habitats, to enable migration towards the Baltic Sea. The reintroduction process for sturgeon is a time-intensive endeavor [3], as these creatures do not reach sexual maturity until about 20 years of age and engage in spawning activities every 2 to 5 years [4].

The F1 generation, representing the initial offspring of the aquaculture-bred Atlantic sturgeon, has now reached reproductive age and has begun its migration from the Baltic Sea back to the rivers. However, the abiotic factors within these waters are an increasingly pressing concern. Specifically, there exists a persistent long-term rise in the temperature of the river water, which strongly correlates with the corresponding increase in air temperatures [5]. This phenomenon manifests itself in a warming rate of approximately 0.30 °C per decade [5]. Seasonal temperature trends in both rivers reveal that during summer, the water experiences the most rapid warming, followed sequentially by spring, autumn, and winter [5]. In addition to the general increase in temperature, the biologically relevant indicator of the annual number of days on which the water temperature exceeds 20 °C has also exhibited synchronous growth with the warming of river water temperatures [5].

Most fish species are considered strict temperature conformers and obligatory poikilotherms, meaning the ambient temperature determines their body temperature. Among the various abiotic factors, temperature is recognized as the main ecological factor [6,7], as it affects the physiological processes described by the Q10 temperature coefficient. Due to their strong correlation, temperature directly impacts the metabolic rate of the fish, consequently affecting their energy balance [7]. The effects of temperature depend on the timing, intensity, and duration of exposure, as well as the rate at which temperature changes occur. While acute, short-term temperature fluctuations can have severe, often detrimental effects on fish physiology, long-term, gradual fluctuations may induce acclimation, leading to alterations in metabolic profiles [7].

Certain organisms possess the ability to partially compensate for the effects of temperature fluctuations on their metabolism. This compensation involves changes at multiple levels of the organization, ranging from biochemical and cellular processes to behavioral responses [8,9,10,11,12,13]. For example, at the cellular level, the density of mitochondria can be regulated. In contrast, at the level of the organism, cardiorespiratory functions can be altered [14]. Many of these adaptations aim to maintain a higher aerobic zone [15] as oxygen levels decrease with rising water temperatures. Consequently, other abiotic factors become critical for fish survival due to the temperature rise.

The genera of sturgeon that exist today date back to their origins about 174 million to 163.5 million years ago and evolved from paleonisiform species that emerged towards the end of the Silurian about 419 million years ago [16]. Throughout their evolutionary history, sturgeons have been exposed to numerous climatic fluctuations. From the Silurian rsp. the middle Jurassic period, temperatures on Earth, including aquatic environments, shifted several times to both extremes, including glacial and interglacial periods [17]. These environmental changes required the adaptation of sturgeons to ensure their survival, ultimately enabling them to be one of the few fossil organisms of the present era. It is plausible to hypothesize that sturgeons possess a substantial physiological capacity that allows them to thrive in various environments, suggesting their potential adaptability to new climatic conditions characterized by higher temperatures.

To comprehend the physiological adaptations of the Atlantic sturgeon, it is essential to perform analyses at the cellular level. Cell models are a valuable tool, as they reduce in vivo studies in endangered fish species and facilitate a more detailed understanding of physiological adaptations through studies at the cellular level. In 2011, Grunow et al. succeeded in establishing a cell line derived from Atlantic sturgeon larvae known as AOXlar7y (*Acipenser oxyrinchus* larvae number 7) through trypsin digestion [18]. This cell line has already been used in studies on temperature adaptation and ecotoxicology [19,20].

In this study, we examined cellular adaptation processes, which include morphological changes, viability, gene expression alterations, and mitochondrial status and plasticity after isolated larvae cells adapt to temperatures of 20 °C, 25 °C, and 28 °C. Our hypothesis assumed that cellular integrity and functionality remain robust over a wide temperature range due to temperature-induced changes in the expression of protective as well as metabolic genes, which could then contribute to the preservation of mitochondrial functions and cell morphology.

The primary objective of this study is to gain further insights into the physiology of the Atlantic sturgeon by transferring data to the animal, particularly with regard to its potential adaptation to aquaculture farming. This understanding could help make predictions about post-release survivability, the viability of offspring, and their ability to contribute to population growth in the wild.

## 2. Materials and Methods

### 2.1. Cell Culture

The AOXlar7y cell line, derived from larvae of Atlantic sturgeon (*Acipenser oxyrinchus*) was obtained from the German Cell Bank for Wildlife (Fraunhofer EMB, Lübeck, Germany) [18]. For cell maintenance and proliferation, the AOXlar7y cells were cultivated in Leibovitz-15 medium (L-15, Gibco, Darmstadt, Germany) with 10% fetal bovine serum (FBS; Pan-Biotech, Adenbach, Germany) and 1% (*v*/*v*) penicillin/streptomycin (P/S; Gibco) at 20 °C. When a cell confluence of 90% was reached, the cells were passaged with a standard trypsinization protocol. For this purpose, the cells were washed with 1× Dulbecco’s phosphate-buffered saline (DPBS; PanBioTech), followed by 2 min trypsinization with 0.1% trypsin/EDTA solution (Gibco) at 37 °C. Trypsinization was stopped by adding at least double the amount of cell culture medium. Cells were centrifuged for 5 min at 130 rcf and sub-cultivated at a ratio of 1:2 to 1:3. Cell numbers and vitality were measured using the Neubauer cell chamber and trypan blue staining.

### 2.2. Temperature Adaption and Cell Growth and Size

In the first step, AOXlar7y cells were analyzed regarding their adaptability to rising temperatures. For this purpose, AOXlar7y cells were transferred to 20 °C (control), to 25 °C (moderate hyperthermia), and to 28 °C (high hyperthermia) 24 h after trypsinization/passaging. For temperature adaptation, the cells were directly transferred from 20 °C to 25 °C. A direct transfer from 20 °C to 28 °C was impossible, as the cells died immediately. Therefore, the cells used in this study were adjusted by increasing the temperature by 1 °C every three to four days until the desired temperature of 28 °C was reached. At this point, cells of each temperature condition were further cultured as described above, with L-15/10% FBS, including 1% P/S, and passaged at 90% confluence using trypsin. The cell experiments were started at least two passages after adaptation. In the course of this research project, the cells were consistently cultivated at the specified experimental temperatures over a period of up to six months. This long cultivation period enabled a comprehensive investigation of the long-term effects of temperature adaptation on the physiological and metabolic properties of the sturgeon larval cell line.

For each experiment, 1.0 × 10^6^ trypsinized cells were cultured in a T75 flask (TPP, Trasadingen, Switzerland) for two days to ensure stable cell adhesion and proliferation. Analyses of the cells were performed after another 48 h, 72 h, and 96 h, and repeated at least six times. Phase contrast microscopic images were taken with a BZ-9000 microscope (Keyence, Neu-Isenburg, Germany), and cell size was measured with the BZ Analyzer 2.2 (Keyence). Cell proliferation and vitality were measured using the Neubauer cell chamber and trypan blue staining.

### 2.3. Staining of Cytoskeleton

A total of 10,000 AOXlar7y cells were plated on chamber slides (LabTek; Thermo Fisher, Dreieich, Germany) and cultured as described above. After the cultivation period, the cells were fixed in 3% paraformaldehyde solution at room temperature for 10 min, permeabilized with 0.1% saponin for 10 min, and washed thrice with PBS. To image the actin filaments (cytoskeleton component) of the cells, Phalloidin Alexa 488 (Thermo Fisher) was added (5 µL in 200 µL PBS) at room temperature for 20 min. Subsequently, the cells were washed thrice with PBS, followed by DAPI staining (Roth, Karlsruhe, Germany; diluted 1:1000 in PBS) for five minutes to visualize the nuclei. The slides were finally rinsed in PBS and aqua dest. and embedded in DAKO fluorescence mounting medium (Roth). Images were taken with a BZ-9000 microscope (Keyence).

### 2.4. Quantitative Real-Time PCR

RNA was isolated from cells grown in a T75 flask (TPP). The RNA was extracted using the RNeasy mini kit (Zymo Research, Freiburg, Germany) according to the manufacturer’s instructions. The RNA concentration was determined by measuring the absorbance at 260 nm with a spectrophotometer (Eppendorf, Hamburg, Germany). The ratio of the absorbances at 260 nm and 280 nm was used to assess the RNA purity. A mass of 1 µg of RNA was reverse transcribed into cDNA with the Biozym cDNA synthesis kit (Biozym, Hessisch Oldendorf, Germany) using the following protocol: 42 °C for 30 min, 85 °C for 10 s, and holding the temperature at 4 °C. Before freezing, the cDNA was diluted to obtain 100 ng aliquots.

Real-time quantifications were performed in duplicates. Samples were mixed with Blue S’Green qPCR Mix Separate ROX (Biozym) containing SYBR green dye and optimized primer pairs (Table 1).

The final PCR protocol involved an initial denaturation step (95 °C, 2 min) followed by 40 cycles of denaturation (95 °C, 5 s) and annealing/extension for 30 s at 60 °C. Melting curve analyses were used to validate the amplification of distinct products. The threshold cycle number (CT), in combination with the 2^−∆CT^ method, was normalized against the housekeeping gene *glyceraldehyde-3-phosphate dehydrogenase* (*gapdh*).

For qRT-PCR, orthologous gene sequences from the NCBI (National Centre for Biotechnology Information) GenBank database were taken, and BLAST searches against the published transcriptome of *Acipenser oxyrinchus* (RefSeq NCBI ASM1318447v1) were performed to identify corresponding sequence fragments. The qPCR primers were designed using Primer3 software (version 4.1.0.; NCBI) for amplifying products with final lengths ranging from 80 to 500 bp. The primers were designed to span the exon–exon junction to ensure an accurate description of our results. In addition, primers were selected solely based on a 100% match with the reference sequence. The following genes were screened for expression: *heat shock genes 70* and *90* (*hsp70* and *hsp90*) for temperature adaptation, *vimentin* (*vim*) for cytoskeleton stability, and *proliferating cell nuclear antigen* (*pcna*) as a proliferation marker. The PCR products were sequenced to evaluate the designed primers, and the obtained sequences were blasted to confirm the genes (Table 1). For this purpose, a cDNA pool was created from the aliquots of all 20 °C, 25 °C, and 28 °C samples and used to generate the respective PCR products. PCR was performed using the HOT FIREPol^®^ blend master mix (Solis Biodyne, Tartu, Estonia). The templates were amplified after 15 min at 95 °C by 35 cycles of the following program: 15 s at 95 °C for denaturation, 45 s for annealing at 60 °C, and 45 s at 72 °C for extension, followed by an additional step for 5 min at 72 °C. The PCR products were purified with the DNA Clean & Concentrator^TM^-5 (Zymo Research, Freiburg, Germany), following the manufacturer’s instructions. Sanger sequencing was performed using the cycle sequencing RR-100, 5× sequencing buffer (Life Technologies, Carlsbad, CA, USA), and primer with a concentration of 3.3 µM. After an initial 1 min denaturation step at 96 °C, 25 cycles with 10 s at 96 °C, 5 s at 50 °C, and 3 min 15 s at 60 °C followed. Cycle PCR products were purified with ethanol precipitation. The SeqStudioTM 8 Flex Genetic Analyzer (Thermo Fischer Scientific, Waltham, MA, USA) was used for capillary electrophoresis.

### 2.5. Seahorse Experiments

AOXlar7y cells were analyzed for mitochondrial and metabolic activity using the Seahorse XF96 (Agilent; Santa Clara, CA, USA) and the corresponding kits (Mito Stress test kit, 103015-100; Glycolysis Stress test kit, 103020; Agilent). For this purpose, the cells cultivated over several passages at different temperatures were seeded at a density of 10,000 cells per well in a Seahorse 96-well cell culture plate (Agilent). Subsequently, the plate was kept for another six hours for cell attachment. Next, before starting the Seahorse experiments, cells were either washed twice with XF Cell Mito Stress Test assay medium containing glucose (1 mM), pyruvate (1 mM), and L-glutamine (2 mM), or a Glycolysis Stress Test assay medium containing 2 mM L-glutamine (starvation media), and then incubated within these media without CO_2_ at 25 °C for 1 h. Meanwhile, a Seahorse sensor plate (Agilent) hydrated with Seahorse XF Calibrant solution (Agilent) was loaded the day before with various inhibitors provided by the kits (oligomycin: final concentration 2 μM; carbonyl cyanide 4-(trifluoromethoxy) phenylhydrazone (FCCP): final concentration 1 μM; rotenone/antimycin A: final concentration 0.5 μM each; glucose: final concentration 10 mM; 2-deoxyglucose: final concentration 50 mM). During the experiment, these reagents were automatically added to the cells, one by one, to pharmacologically block or stimulate different parts of the mitochondrial electron transport chain or glycolysis to measure the oxygen consumption rate (OCR) or extracellular acidification rate (ECAR). Upon completion of the assay, the medium was discarded, and the plate was frozen at −70 °C before determining the cellular protein content per well. These measurements were used to normalize the Seahorse data and calculate different parameters of the cellular bioenergetics profile according to Agilent’s software (WAVE, Toronto, ON, USA, version 2.6.1), Table 2.

### 2.6. Measurement of Protein Content and Normalization

In order to exclude possible variations due to the temperature-dependent growth rates of AOXlar7y cells, a determination of the protein contents per well was performed after the Seahorse experiments. For this purpose, the protocol described by Wanka et al. was used [21]. The frozen Seahorse cell culture plates were thawed, and the remaining cells were lysed in 12 μL of RIPA buffer containing 33.3 mmol/L Tris, 3.33 mmol/L EDTA, 100 mmol/L NaCl, 6.67 mmol/L K_2_HPO_4_, 6.67% glycerol, 0.67% TritonX-100, 1 mmol/L NaVO4, 20 mmol/L NaF, 0.1 mmol/L PMSF, 20 mmol/L 2-phosphoglycerat, and a protease inhibitor cocktail (Roche Diagnostics, Mannheim, Germany). Then, 50 μL of the Bradford reagent (Roth) was added to 50 μL of the 1:10 diluted sample. The protein content was measured according to the manufacturer’s instructions.

### 2.7. Statistical Analysis

All statistical analyses were performed using GraphPad Prism software, version 9.3.1. (La Jolla, CA, USA). The data are presented as mean ± SD (standard derivation). The group comparisons were made using two-way ANOVA with Tukey’s multiple comparison test of 6 individual experiments. Results were considered statistically significant at *p* < 0.05.

## 3. Results

### 3.1. Characteristics of the Cells

**Figure 1:**
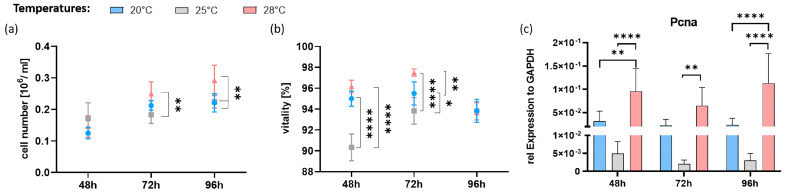
Characteristics of AOXlar7y cells cultured at different temperatures. (**a**) Cell number, (**b**) vitality and copy numbers of the genes, (**c**) *pcna* expression (proliferation marker) normalized to the expression level of the housekeeping gene *gapdh*. Cultures were performed at 20 °C (control; blue), 25 °C (moderate hyperthermia; grey), and 28 °C (high hyperthermia; red) for 48 h, 72 h, and 96 h. Data represent mean ± SD of six individual experiments. Statistical significance (two-way ANOVA) as indicated: * *p* < 0.05, ** *p* < 0.01, **** *p* < 0.0001).

The analysis revealed increasing cell numbers over time at all three temperatures (Figure 1a). While no cell number differences were detectable after 48 h of culture, the cells cultured at 28 °C were significantly higher in number than that of the 25 °C group after 72 h, and significantly higher than those of the two colder temperatures after 96 h (all *p* < 0.001). At all temperatures and cultivation times, the cell vitality was above 90% (Figure 1b). The lowest vitality was present in the 25 °C group after 48 h. After 72 h, the vitality was still the highest in the 28 °C group (97.5%), followed by the 20 °C group (95.5%) and the 25 °C group (93.8%). The cell number results correlated with the gene expression analysis of *pcna*, which serves as a proliferation marker (Figure 1c). Analogous to cell numbers, the increase in culture temperature from 20 °C to 25 °C did not affect relative *pcna* expression levels at all three time points, although a tendency to decline was observed. Compared to the 20 °C control group, *pcna* expression in the 28 °C group increased significantly at 48 h and 96 h incubation. As *pcna* expression tended to decrease in the 25 °C group, the expression levels in the 28 °C group were significantly higher than the 25 °C group at all time points (*p* < 0.01, Figure 1c).

**Figure 2:**
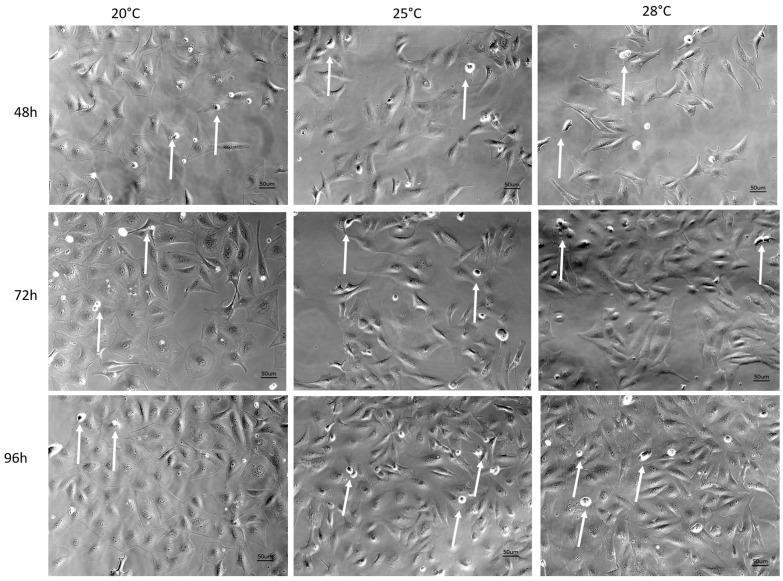
Morphological appearance of AOXlar7y cells 48 h, 72 h, and 96 h after culture at 20 °C (control), 25 °C (moderate hyperthermia), and 28 °C (high hyperthermia). Arrows indicate cells undergoing division.

Phase contrast microscopy allows for the morphological analysis of cells, i.e., their appearance and adhesion to the culture plate, as well as the presence of proliferating, detached cells (Figure 2). The images show that the cell densities increased over time in all groups studied, and proliferating cells were present. In addition, cells of the control group appeared flatter compared to the 25 °C and 28 °C groups.

### 3.2. Cytoskeleton

Cells can be very heterogeneous in shape due to their intrinsically viscoelastic cytoskeleton, which allows them to deform on demand [22]. Therefore, their structural organization and mechanical properties are closely related to their optical appearance [23].

**Figure 3:**
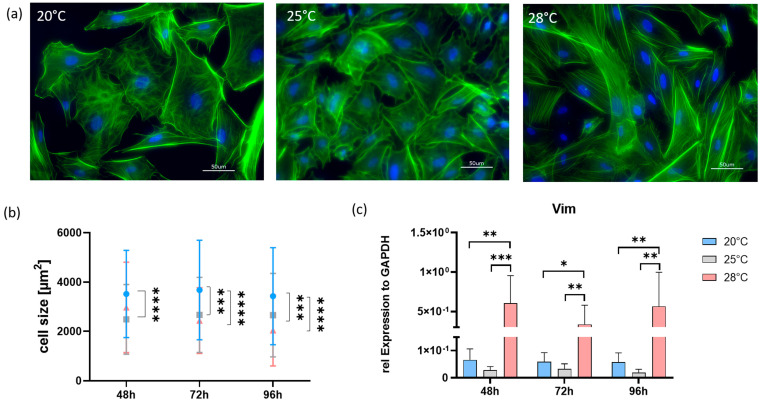
Examination of the cytoskeleton of AOXlar7y cells. (**a**) Immunofluorescence staining of actin filaments of the cells after 96 h of cultivation. (**b**) Cell size measurement of attached cells after 48 h, 72 h, and 96 h of cultivation. (**c**) Copy numbers of the *vimentin* (*vim*) gene normalized to the expression of *gapdh*. Cell culture was performed at 20 °C (blue), 25 °C (grey), and 28 °C (red) for 48 h, 72 h, and 96 h. Data represent mean ± SD of six individual experiments. Statistical significance (two-way ANOVA) as indicated: * *p* < 0.05, ** *p* < 0.01, *** *p* < 0.001, **** *p* < 0.0001.

Immunofluorescence staining with the fluorescent phalloidin that binds to filamentous (F)-actin was used to determine the effects of temperature on the nature of the cytoskeletal structures. The distribution of F-actin was found to be different in the cells, depending on the cultivation temperature (Figure 3a). At 20 °C and 25 °C, the F-actin filaments were thin and distributed throughout the AOXlar7y cells. At and near the cell periphery, the filaments seemed to become slightly thicker. In cells cultured at 28 °C, a change was evident. Here, F-actin filaments were found in the cells rather linearly, and an increased accumulation of F-actin seemed to occur in the cell periphery.

Analyses of the attached cells indicated a decrease in cell size due to temperature increase (Figure 3b). Compared to the control cells at 20 °C, a temperature increase of 8 °C resulted in significantly smaller cells after 72 h and 96 h. In contrast, cells incubated at 25 °C showed significantly smaller cell sizes after 48 h, 72 h, and 96 h (all *p* < 0.0001). The gene expression analysis of *vim*, a cytoskeleton marker, revealed significantly higher expressions in the 28 °C group compared to the 20° (*p* < 0.05) and 25 °C (*p* < 0.01) groups at all three time points. No significant differences were found between the 20 °C and 25 °C groups (Figure 3c).

### 3.3. Stress Adaptation Analyses

**Figure 4:**
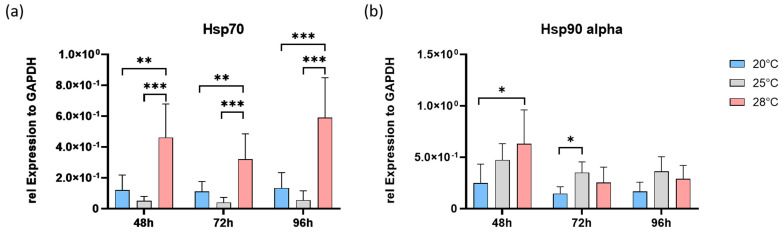
Temperature-dependent gene expression profiles of *heat shock proteins 70* (*hsp70*) and *90 alpha* (hsp90a) in AOXlar7y cells. (**a**) Copy numbers of the *hsp70* and (**b**) *hsp90a* genes, each normalized to the housekeeping gene *gapdh*. AOXlar7y cells were exposed to 20 °C (blue), 25 °C (grey), and 28 °C (red) for 48 h, 72 h, and 96 h of cultivation. Data represent mean ± SD of six individual experiments. Statistical significance (two-way ANOVA) as indicated: * *p* < 0.05, ** *p* < 0.01, *** *p* < 0.001.

In the next step, the expressions of the *heat shock proteins 70 (hsp70*) and *90 alpha* (*hsp90a*) were analyzed. The profile of *hsp70* expression was very similar to that of *vim* (Figure 3c). The expression of *hsp70* remained constant over time in the control group (Figure 4a). There were no significant differences between cells cultured at 20 °C and 25 °C for all three time points. However, *hsp70* expression in the 28 °C group was always significantly higher than that of the cells at lower temperatures (*p* < 0.01).

In contrast, the expression of *hsp90a* in cells cultured at 28 °C was significantly higher only after 48 h (*p* < 0.05; Figure 4b). After 72 h and 96 h, no differences were detectable compared with cells at lower temperatures. In the control and 25 °C groups, *hsp90a* expression remained constant over time. However, significantly higher expression of *hsp90a* was detected in the 25 °C group compared to the control group after 72 h of incubation. After extending the culture to 96 h, there were no differences in *hsp90a* expression between the groups studied.

**Figure 5:**
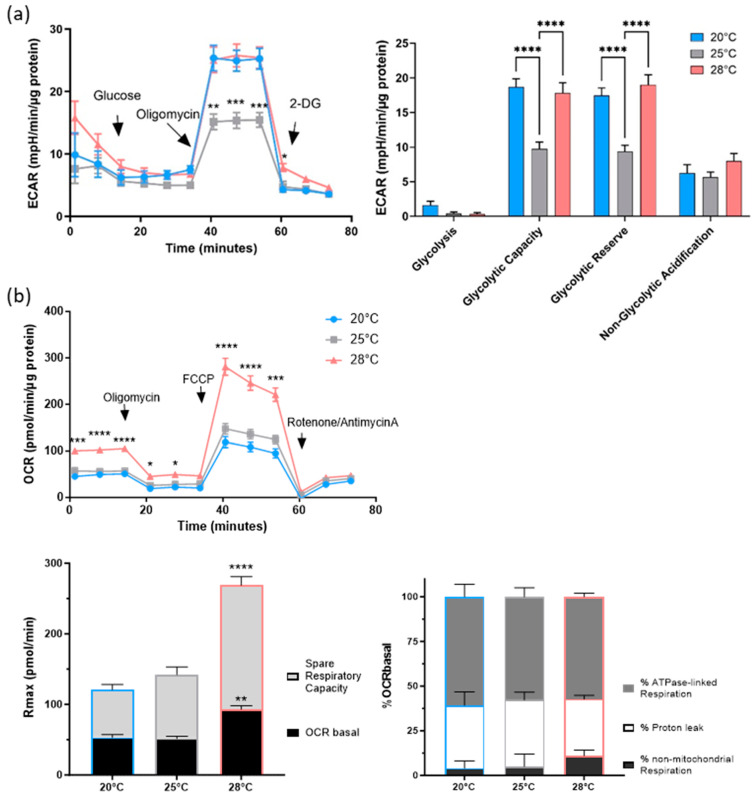
Bioenergetic parameters of AOXlar7y cells incubated at 20 °C (blue), 25 °C (grey), and 28 °C (red). Cells were cultivated at a density of 10,000 cells per well in ten technical replicates for six hours for cell attachment before performing the (**a**) glycolysis stress test and (**b**) Mito Stress test. All measured values were normalized to protein content. (**a**) The extracellular acidification rates (ECAR) of the cells were measured under starvation and after glucose, oligomycin, and 2-deoxyglucose (2-DG) administration (indicated by arrows). Glycolysis, glycolytic capacity and reserve, and non-glycolytic acidification were calculated using normalized ECAR values obtained with WAVE software (version 2.6.1). (**b**) The oxygen consumption rates (OCR) were first measured under basal conditions and then after applying various inhibitors of mitochondrial respiratory chain components (oligomycin, FCCP, and rotenone/antimycin A) at the time points indicated by an arrow. The maximal respiration (Rmax) is composed of OCRbasal and spare respiratory capacity. The percentages of ATP synthase-linked respiration, proton leak, and non-mitochondrial respiration from basal respiration were calculated using normalized OCR values obtained with WAVE software (version 2.6.1). Two-way ANOVA and Šídák’s multiple comparisons test were performed for statistical analysis. * *p* < 0.05, ** *p* < 0.01, *** *p* < 0.001, **** *p* < 0.0001, (**a**) 20 °C and 28 °C vs. 25 °C and (**b**) 20 °C and 25 °C vs. 28 °C.

In a further study, the AOXlar7y cells incubated at different temperatures were investigated and characterized for their metabolic function using Seahorse technologies. We showed that adding glucose to glucose- and pyruvate-starved AOXlar7y cells did not induce glycolysis (Figure 5a). The oligomycin-induced inhibition of mitochondrial ATP production resulted in greater glucose utilization by the control (20 °C) and 28 °C groups compared to the 25 °C group. Consequently, both the glycolytic capacity and reserve were higher in these cells than in cells incubated at 25 °C. Non-glycolytic acidification was unaffected by incubating the cells at different temperatures.

Measurements of mitochondrial function in AOXlar7y cells exposed to different temperatures demonstrated significantly higher basal respiration and higher maximal respiration (Rmax) in 28 °C-incubated cells compared to the other groups (both *p* < 0.001; Figure 5b, upper left). When the Rmax was split into basal OCR (OCRbasal) and spare respiratory capacity, the ratio was 35% to 65% in the 28 °C group (Figure 5b, lower left), similar to the ratio of the 25 °C group. Interestingly, this ratio was 45% OCRbasal and 55% spare respiration capacity in the control group. Thus, the 25 °C- and 28 °C-exposed cells had almost twice the spare respiratory capacity of basal respiration, while the control cells at 20 °C exhibited only about 1.2 times the spare respiration capacity.

However, the proportion of ATP synthase-linked respiration, proton leak, and non-mitochondrial respiration as components of OCRbasal did not show significant differences between the groups (Figure 5b, lower right). Regardless of the culture temperature, all cells mainly used oxidative phosphorylation (about 58%) to generate the required ATP. In contrast, about 4–5% of total oxygen consumption was due to non-mitochondrial respiration in cells incubated at 20 °C and 25 °C, and 11% in cells incubated at 28 °C (not significant).

## 4. Discussion

Environmental temperature directly affects the physiology and behavior of ectothermic organisms, including fish. In this study, a cell line from Atlantic sturgeon larvae (AOXlar7y) was exposed to three different temperatures to track their physiological adaptations at the cellular level. The study shows that AOXlar7y cells had significantly increased proliferation rates after adapting to a temperature of 28 °C. This was confirmed by increases in cell numbers, as well as *pcna* expression levels, especially after 48 and 96 h of culture. It is known that within the tolerance range, temperature increases of 10 °C increase the reaction rate by a factor of 2 to 3 (thermal sensitivity quotient (Q10)) [24]. This could explain why the cells exposed to a culture temperature of 25 °C did not react, meaning that the temperature increase in vitro was insufficient to induce adequate responses. Another reason could be the significantly reduced vitality of the 25 °C exposed cells in the first days of culture, which did not allow the cells to proliferate. After 96 h in culture, we noticed that the vitalities were similar between the groups. There were no longer any significant differences. The decline in vitality in the 28 °C group may be due to the increased cell numbers, which may have led to alterations in the culture medium, such as accumulation of metabolic end products or changes in pH or contact inhibition by overgrowth in the well. However, the vitality data from the trypan exclusion test must be carefully evaluated, as the uptake of trypan blue into the cytosol requires impaired cell membrane integrity [25]. This applies to necrotic cells, while early apoptotic cells with an intact membrane are not detectable. Overall, the study shows that the AOXlar7y cells can tolerate higher temperatures for several days and even months. In addition, the selected cell lines can also be maintained by freezing and thawing without any apparent difference in viability.

However, more in-depth analyses of cell physiology show that high temperature seems to influence cell structure and metabolism. For this purpose, filamentous actin (F-actin) distribution, which plays a crucial role in the cytoskeleton structure by imparting intrinsic viscoelasticity and significantly influencing the mechanical properties of cells [26], was analyzed first. By means of phalloidin staining, characteristic ventrally oriented fibers could be identified, especially in 28 °C cultured cells, which could be so-called stress fibers [27]. Actin stress fibers interact with the plasma membrane of cells and are involved in cell structure, migration, proliferation, and signaling pathways [27]. In addition to the F-actin network, there are vimentin-based intermediate filaments (VIP) that expand from the cell membrane to the nucleus [28]. VIP are connected to the actin cytoskeleton and organelles and interact with various signaling proteins, including extracellular signal-regulated kinase (ERK), rho-associated protein kinase (RhoK), and 14-3-3 proteins. All of this suggests that vimentin provides cells with a protective or pro-survival function and may be involved explicitly in heat stress tolerance [29].

In addition, the cells were significantly reduced in size when the temperature was increased by both 5 °C and 8 °C. Cell size is an important feature for understanding the thermal biology of ectotherms, as the regulation and consequences of cell size appear to be temperature-dependent [30,31,32,33]. Thus, the surface-to-volume ratio is greater in smaller cells than in larger cells of the same shape. Therefore, it can be calculated that the 28 °C cells have a higher diffusion rate due to their increased surface area [33]. In this context, the metabolism of smaller cells is also energetically complex due to this larger surface-to-volume ratio since lipid turnover and maintenance of electrochemical gradients on cell membranes are more expensive [34,35]. Therefore, reducing the cell size can contribute to better cell survival.

The expression of heat shock proteins (HSPs) is a good indicator of heat stress, as protection against heat at the cellular level involves the synthesis of chaperone proteins [36,37]. In fact, HSPs are highly conserved in the animal kingdom [38]. They are responsible for preventing protein aggregation, refolding stress-denatured proteins, and reducing cell death through apoptosis [39]. An association between the ability to upregulate HSPs and the development of thermal tolerance has also been found in aquatic species [40,41,42,43], suggesting a potential ecological relevance of HSPs in the context of global warming. In our study, cells cultured at 28 °C showed higher mRNA expression of *hsp70* at all time points investigated. The upregulation of *hsp70* expression is part of a cellular defense response [44,45] because HSP70 protein binds to unfolded or misfolded proteins, preventing their aggregation and promoting their correct folding [46]. In summary, the increased expression of *hsp70* in response to elevated temperature is a protective mechanism that cells use to mitigate the harmful effects of heat stress. By supporting protein folding and preventing the accumulation of damaged proteins, HSP70 helps maintain cellular homeostasis and ensures cell survival under thermal stress conditions [47].

Interestingly, the expression of *hsp90a* was initially increased and decreased to basal values when the cells were exposed to a temperature of 28 °C for 72 and 96 h. HSP90a is known to have a cytoprotective effect by being involved in recovery before and adaptation to cellular stress. This effect is realized by controlling cellular signaling cascades, restoring global protein synthesis, and coordinating cellular repair [48,49,50,51]. Due to the time-dependent differences in *hsp90a* expression in the 28 °C-exposed cells described above, we speculate that the cells were subjected to greater stress than the control cells due to detachment and transfer to the experiment but recovered with a time lag.

Next, we hypothesized that an increase in temperature could lead to metabolic or mitochondrial reprogramming in the AOXlar7y cells since temperature-dependent metabolism has also been described for other species and cell types [52,53,54]. Therefore, we used the Seahorse XF technology, which is widely used to analyze cellular metabolism by measuring OCRs and ECARs in living cells in real-time [52]. To our knowledge, this study is the first to investigate temperature-induced changes in the dominant energy-supplying pathways (glycolysis or mitochondrial respiration) of sturgeon larvae cells adapted to temperature increases.

The glycolytic function of larval cells was determined using the glycolysis stress test, which measures the extracellular acidification rate (ECAR), reflecting the release of protons in symmetry with the glycolysis product lactate into the extracellular environment (pyruvate to lactate catalyzed by lactate dehydrogenase), but also the protons released from CO_2_ and H_2_O by carbonic anhydrase-induced formation of HCO^3−^ and H^+^ after CO_2_ export [55]. Mitochondrially produced energy was indicated by the Mito Stress Test assay, which measures the oxygen consumption rate (OCR). Along the electron transport chain, which consists of four complexes (complex I, II, III, and IV), electrons are transferred to oxygen and form a water molecule with two protons from the matrix, while a proton gradient is established that is used by ATP synthase to generate ATP.

From the ECAR and OCR data, we conclude that the sturgeon cells were able to obtain ATP from both glycolysis and mitochondria-derived oxidative phosphorylation but prefer mitochondria-dependent ATP production. The glycolytic function of the larvae cells revealed that the cells hardly performed glycolysis independent of culture temperature, confirming previous reports regarding reduced glucose turnover in fish [56]. Since no differences in non-glycolytic acidification or rates of glycolysis were observed in all three groups, we hypothesized that the temperature increase did not affect basal glycolysis and basal bioenergetics. However, decreasing glycolytic capacity and glycolytic reserve in 25 °C exposed cells suggest a reduced potential to increase ATP production via glycolysis under stress or other physiologically energy-demanding situations. The data from the Mito Stress Test clearly showed increased basal respiration, maximal respiration, and spare respiratory capacity in larvae cells exclusively exposed to 28 °C. Interestingly, the percent composition of the basal OCR of cells exposed to 28 °C, in terms of ATPase-linked respiration, proton leak, and non-mitochondrial respiration, did not differ from the composition of cells cultured at 20 °C or 25 °C. However, the increases in basal and maximal OCR of the 28 °C cells can be interpreted as a change in mitochondrial ATP synthesis due to changes in ATP-demanding processes, mitochondrial content, or activities of rate-controlling enzymes such as complex I [57]. The temperature increase of 8 °C resulted in twice the spare capacity as basal respiration, demonstrating increased cellular flexibility, response to energy requirements, and adaptability [56]. To clarify the underlying effects, detailed analyses of mitochondrial number, subtype, and membrane potential, as well as expression studies of mitochondrial and glycolytic proteins, could provide further insights into the results obtained.

## 5. Conclusions

In summary, AOXlar7y cells adapted to 28 °C exhibited increased mitochondrial adaptability, flexibility, and plasticity combined with increased proliferation and *hsp70* expression; however, their glycolytic capacity remained unaffected. The higher basal respiration of mitochondria at this temperature suggests that (1) there is a higher ATP demand, which could be due to increased proliferation rate and protection during heat stress, all ATP-dependent processes, and (2) that this demand is met by ATPase-dependent ATP production. However, incubating the cells at 28 °C also led to a better capability to respond to energetic demands, as indicated by their elevated spare respiratory capacity. In contrast, no changes in basal respiration and mitochondrial respiratory capacity of the AOXlar7y cells at 25 °C were observed compared to controls, suggesting no higher ATP demand at this temperature. Proliferation and glycolytic capability to respond to an energetic ATP demand were significantly reduced. These could be directly related, as dependence between glycolysis inhibition and proliferation inhibition has been demonstrated [58]. The results of this study indicate that sturgeon cells have good and rapid metabolic adaptability to higher temperatures, which should further be analyzed in future studies to understand the mechanism of ROS and further metabolomics pathways. Overall, the in vitro analyses at the cellular level provide a prerequisite for a better understanding the environmental threats to Atlantic sturgeon in particular and to fish in general.

## Figures and Tables

**Table 1:** Gene-specific primers used in this study.

Gene Symbol	Official Names	Genbank ID	NCBI Reference Sequence	Forward Primer (5′-3′)	Reverse Primer (5′-3′)	qPCR Product (bp)
Housekeeping Gene						
*gapdh*	*glyceraldehyde-3-phosphate dehydrogenase*	SCEB01214188.1.1	XM_034042013.3	ACACCCGCTCATCAATCTTT	AGGTCCACGACTCTGTTGCT	80
Target Gene						
Proliferation						
*pcna*	*proliferating cell nuclear antigen*	MK077520.1	XM_059013649.1	GCTGTGACGATCGAGATGAA	AACCAGAGCACACATGCTG	215
Cytoskeleton						
*vim*	*vimentin*	AJ493266.1	XM_034058632.3	GATTTCGCCTTGTCCGATGC	TTGGTGGTGCGTTTTCCCTT	350
Stress-related genes						
*hsp70*	*heat shock protein 70*	JN098420.1	XM_033996031.3	CCCGTGGAGAAGTCC	CCCGTTGAAGAAATCCTG	123
*hsp90a*	*heat shock protein 90alpha*	JN700180.1	XM_034018059.3	GGTCATCTTGCACCTGA	TTCTGCTTCATCATCGCTG	250

**Table 2:** Respiration indices and their means of calculation.

Index	Calculation of Index Values
Mito Stress Test	
Basal respiration	Baseline OCR—non-mitochondrial respiration
ATP production	Basal respiration—oligomycin OCR
Maximal respiration	FCCP respiration—non-mitochondrial respiration
Space capacity	FCCP respiration—basal respiration
Non-mitochondrial respiration	OCR with rotenone/antimycin A treatment
Glycolysis Stress Test	
Glycolysis	Glucose ECAR—starvation ECAR
Glycolytic Capacity	Oligomycin ECAR—starvation ECAR
Glycolytic Reserve	Glycolytic Capacity—Glycolysis
Non-Glycolytic Acidification	Starvation ECAR

## Data Availability

All relevant data are provided within the manuscript.
